# The Reduction of Left Ventricle Ejection Fraction after Multi-Vessel PCI during Acute Myocardial Infarction as a Predictor of Major Adverse Cardiac Events in Long-Term Follow-Up

**DOI:** 10.3390/ijerph192013160

**Published:** 2022-10-13

**Authors:** Michał Chyrchel, Tomasz Gallina, Rafał Januszek, Oskar Szafrański, Monika Gębska, Andrzej Surdacki

**Affiliations:** 1 Second Department of Cardiology, Jagiellonian University Medical College, ul. Jakubowskiego 2, 30-688 Kraków, Poland; 2 Student Scientific Group at the Second Department of Cardiology, Jagiellonian University Medical College, ul. Jakubowskiego 2, 30-688 Kraków, Poland; 3 Department of Cardiology, District Hospital, ul. Jagiellońska 36, 97-500 Radomsko, Poland

**Keywords:** acute myocardial infarction, complete revascularisation, left ventricle ejection fraction, multi-vessel coronary disease, percutaneous coronary intervention

## Abstract

Background: Revascularisation strategy in patients with multi-vessel coronary disease and acute myocardial infarction (AMI) remains challenging. One of the potential treatment options is complete percutaneous revascularisation during index hospitalisation. This strategy could positively influence left ventricle ejection fraction (LVEF). Aim: To investigate the long-term changes in LVEF and clinical outcome among patients with AMI after complete coronary revascularisation (CCR). Methods: Records of 171 patients with a diagnosis of AMI and multi-vessel coronary artery disease (CAD) on index angiography, in whom CCR was performed as a staged procedure during initial hospitalisation, were analysed. Clinical data were collected from in-hospital medical records and discharge letters. Cardiac ultrasound (CU), with particular assessment of LVEF, was performed one day before discharge. Follow-up (FU) CU was collected from the out-patient department at least six months ± one week after discharge. Follow-up data, including major adverse cardiac events (MACE), were collected during follow-up visits by telephone. Depending on the LVEF change during the follow-up period, patients were divided into two groups. Patients with a decrease in the LVEF (D-LVEF group) were compared with patients with no changes (preserved) or improvement regarding LVEF (P/I-LVEF). Results: The median duration of the follow-up was 19 months (14–24 months). The median change in LVEF during observation was –5.0p% (IQR (–7.0)–(-2.75p.%)) in the D-LVEF group and +4.0% (IQR 1.0–5.0p%) in the P/I-LVEF group. Among patients in the P/I-LVEF group, there was a sub-group of patients with no change in LVEF (28 patients), and one demonstrating improvement in LVEF (104 patients). In the subgroup of patients with improved LVEF, the median change in LVEF was 4.5p% (IQR 2–6.25p%). Among patients with decreasing LVEF, there was a significantly higher risk of MACE (15 vs. 2.3%, *p* = 0.031), especially non-fatal AMI (10 vs. 0%, *p* = 0.017). We found the following among predictors concerning increased risk of MACE occurrence: urgent PCI (*p* = 0.004), hospitalisations regardless of cause (*p* = 0.028), EF worsening (*p* = 0.025), fasting glucose serum concentration (*p* = 0.024) and fasting triglyceride serum concentration (*p* = 0.027). Conclusions: Complete revascularisation (CR) at baseline (one stage) in patients with AMI and multi-vessel disease is associated with LVEF improvement and MACE rate reduction. Patients with worse LVEF have poor clinical outcome and a higher rate of MACE.

## 1. Introduction

Complete revascularisation (CR) could be considered during index hospitalisation in patients with acute myocardial infarction (AMI) and multi-vessel coronary disease (MVD) [1,2]. Multi-vessel percutaneous coronary angioplasty (PCI) in patients presenting with AMI can be performed during one procedure, as a two-stage procedure during the same hospitalisation or can be completed as a multi-staged procedure, i.e., a few weeks after first PCI. Strategy selection depends on clinical characteristics of the patient, comorbidities and also local protocols at different cardiology centres, as well as reimbursement aspects. Scientific evidence supports multi-vessel angioplasty strategy (one session or staged procedure), which seems to reduce the need for urgent revascularisation during follow-up and has a beneficial influence on other clinical endpoints [3,4]. Left ventricle ejection fraction (LVEF) is a strong haemodynamic factor influencing the clinical outcome of patients with AMI. It was shown that reduced LVEF is associated with increased mortality in patients with ST-elevation myocardial infarction (STEMI) [5]. Complete revascularisation during index hospitalisation could result in LVEF improvement in patients with initially impaired LVEF or could have help to maintain the similar EF values in patients with initially preserved LVEF. Another potential beneficial effect of CR is the reduction in angina pectoris intensification during follow-up.

In the present study, we investigated changes in the LVEF and clinical outcome if patients with AMI after percutaneous CR in long-term follow-up.

## 2. Methods

### 2.1. General Characteristics

We have retrospectively analysed the records of 171 patients from December 2017 to January 2019 with a diagnosis of acute coronary syndromes: ST-segment elevation myocardial infarction (STEMI) or non-ST segment elevation myocardial infarction (NSTEMI) and multi-vessel coronary artery disease (CAD) on index angiography, in whom complete PCI was performed as a staged procedure during initial hospital stay at one of two catheterisation laboratories (CathLabs). Cardiogenic shock patients were excluded from the study. Other exclusion criteria included: age below 18 years, haemodynamic instability (Killip class 3 or 4), critical left main coronary artery stenosis, mechanical complications of AMI, prior coronary artery by-pass grafting (CABG), current chemotherapy, advanced renal failure or renal-replacement therapy.

### 2.2. Data Collection and Additional Calculations

Clinical, demographic and biochemical patient characteristics were collected from in-hospital medical records and discharge letters. Echocardiographic examination with particular assessment od LVEF was performed one day before discharge. Follow-up echocardiography was performed in the out-patient department at least six months ± one week after discharge. Follow-up data were collected during follow-up visits by telephone. The follow-up visit included information on the occurrence of: major adverse cardiac events (MACE), which consisted of death, non-fatal AMI and non-fatal stroke or TIA, urgent PCI, planned PCI, CABG and other hospitalisations regardless of cause. Patients were also asked about inclusion in cardiac rehabilitation programmes, haemorrhagic complications (gastro-intestinal, urine tract, central nervous system) and local complications (associated with vascular access). Actual severity of angina pectoris was assessed using CCS. Questions also concerned actual reception and type of DAPT (Dual Antiplatelet Therapy). Despite the change in LVEF during the follow-up period, patients were divided into two groups. Patients with any decrease in LVEF (D-LVEF group) were compared with patients demonstrating no change (preserved) or improvement in LVEF (P/I-LVEF group).

### 2.3. Statistical Analysis

All data were tested with the Shapiro–Wilk test for normality. Continuous data were presented as means and standard deviations (SD) for parametric variables, or the median and interquartile range (IQR) for non-parametric variables, while nominal data were shown as absolute amounts and percentages. To compare data between groups, the Student’s *t*-test, Wilcoxon test, Mann–Whitney U test and the Chi square test were used appropriately. The significance level was set at *p* < 0.05. We performed univariable logistic regression analysis concerning the potential risk factors of MACE occurrence. We included all potential predictors of MACE into the analysis. However, we were not able to construct a multi-variable logistic regression model due to the low number of MACEs. Statistical analysis was performed using jamovi 1.2.27 software.

## 3. Results 

### 3.1. Baseline Characteristics

The follow-up was completed in 88.9% (152/171) of patients. Among the study population, there were 99 (65.1%) men and 53 (34.9%) women. The median duration of the follow-up was 19 months (14–24 months). The mean age was 67.3 years. Initial diagnosis during index hospitalisation—STEMI (n = 72; 47.4%); NSTEMI (n = 80; 52.6%). The median duration of hospitalisation was 6 days (IQR 5–8). There were no significant differences between the D-LVEF and P/I-LVEF groups (Table 1). Patients in both groups suffered from many comorbidities. The prevalence of the most significant comorbidities and coronary artery disease risk factors was also presented in Table 1. As shown below, the prevalence of diabetes was significantly more frequent in the D-LVEF group (50% vs. 27.3%, *p* = 0.038), while patients in the D-LVEF group also had higher body mass index (BMI) (32.1 vs. 27.6 kg/m^2^, *p* = 0.032).

### 3.2. Changes in LVEF and Follow-Up

Every patient during echocardiographic examination had LVEF assessed. During statistical analysis, the LVEF at the time of discharge was compared with that from out-patient department examination. Patients with any decrease in the level of LVEF (D-LVEF group) were selected from the study population and compared with patients showing improvement, or at least with no change, in LVEF (P/I-LVEF group).

**Figure 1:**
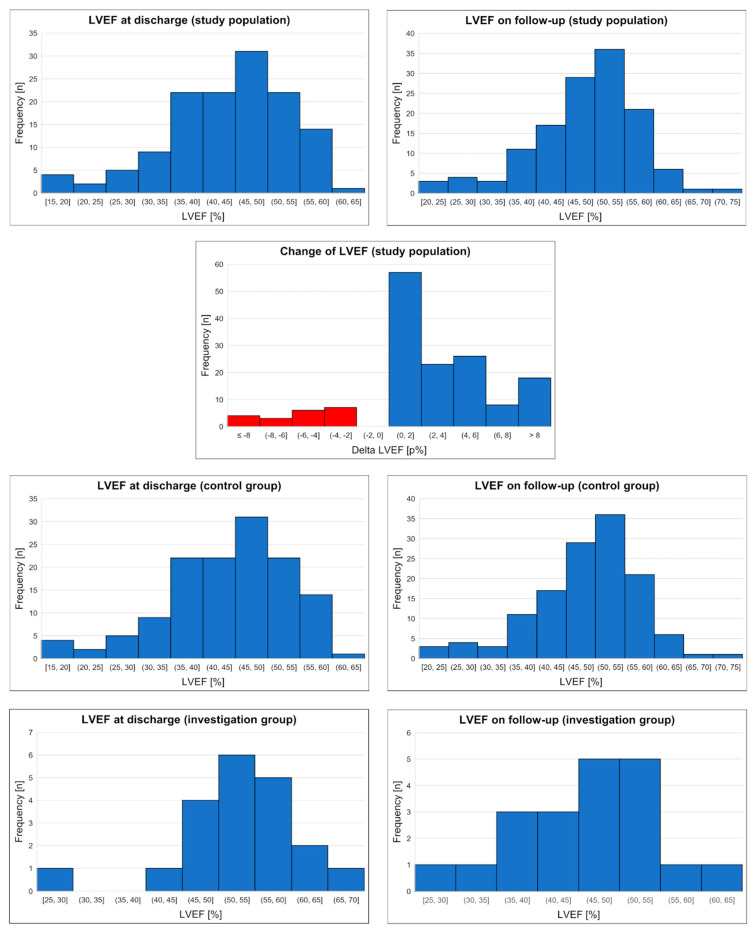
Histograms of LVEF among study populations, P/I-LVEF group and D-LVEF group. LVEF (left ventricle ejection fraction).

We observed interesting phenomena that patients with worsening LVEF during the follow-up had significantly higher LVEF at the time of discharge (54.5 vs. 46%, *p* < 0.001). The median change in LVEF during observation was –5.0p% (IQR (–7.0)–(–2.75p.%)) in the D-LVEF group and +4.0p% (IQR 1.0–5.0p%) in the P/I-LVEF group. Among patients from the P/I-LVEF group, there was a subgroup of patients lacking change in LVEF (28 patients) and a subgroup of patients with improvement noted in LVEF (104 patients). In the subgroup of patients with improved LVEF, the median change was 4.5p% (IQR 2–6.25p%). All data are presented in Table 2. Detailed data for LVEF and its changes were also presented in histograms—Figure 1.

### 3.3. MACE Prevalence

The main outcome in our study was composite, defined as death, non-fatal MI, non-fatal stroke or TIA. These outcomes were classified as MACE. We also analysed other adverse events during the follow-up as urgent PCI, planned PCI or any hospitalisation regardless of cause. Statistical analysis revealed: death rate (n = 2; 1.3 %), non-fatal AMI (n = 2; 1.3%), urgent PCI (n = 4; 2.6%), planned PCI (n = 7; 4.6 %), non-fatal stroke (n = 2; 1.3%) and that there was no reported event of TIA during re-hospitalisation (n = 38; 25.0%). We also compared the prevalence of MACE and other adverse events between the D-LVEF and P/I-LVEF groups. Among patients with worsening LVEF, there was a significantly higher risk of MACE (15 vs. 2.3%, *p* = 0.031), especially non-fatal AMI (10 vs. 0%, *p* = 0.017). In addition, the risk of hospitalisation, regardless of cause, was significantly higher in this group (45 vs. 22%, *p* = 0.027). During phone visits, all patients were asked about haemorrhagic complications connected with DAPT and local complications associated with PCI performed during index hospitalisation. There were no significant differences between the groups. All data are presented in Table 3.

### 3.4. Univariate Analysis of MACE Predictors

Among the predictors concerning increased risk of MACE occurrence, we found: urgent PCI (*p* = 0.004), hospitalisations regardless of cause (*p* = 0.028), EF worsening (*p* = 0.025), fasting glucose serum concentration (*p* = 0.024) and fasting triglyceride serum concentration (*p* = 0.027). Only HDL-cholesterol serum concentration was associated with reduced risk of MACE occurrence (*p* = 0.031). All data are presented in Table 4. 

### 3.5. Diabetes Prevalence and Its Influence on MACE

As shown in Table 1, among patients with worsening LVEF, there was a higher frequency of diabetes mellitus compared to the P/I-LVEF group—50 vs. 27.3%, *p* = 0.039. However, among the study population, diabetes mellitus was only associated with a higher frequency of hospitalisation compared to non-diabetic patients (43.5% vs. 17%, *p* < 0.001). A higher hospitalisation rate was observed among patients with diabetes and decreasing LVEF (60%) than among patients with diabetes from the P/I-LVEF group (38.9%) or among non-diabetic patients with worsening LVEF (30%), while the least risk of hospitalisation was observed among non-diabetic patients with no decreasing LVEF (15.6%). This difference was significant at level of *p* = 0.002 in the Chi square test. There were no other significant differences between the diabetic and non-diabetic patients. All data are presented in Table 5.

### 3.6. Severity of Angina Pectoris at Follow-Up; CCS Class Analysis

Severity of angina pectoris was assessed using the CCS classification. The majority of patients presented minor or minimal symptoms; n = 116 (78.4 %) in Class I and 26 (17.6 %) in CCS II. Five patients (3.4 %) were classified into CCS III while one patient was attributed to class IV (0.7 %). 

There were significant differences between the D-LVEF and P/I-LVEF groups (Table 6). Patients with a reduction in LVEF during the follow-up were significantly more often classified into class III and significantly less often to class I.

### 3.7. Subgroup Analysis: STEMI vs. NSTEMI

STEMI patients, in comparison to NSTEMI, were: younger (65.5 vs. 69 years; *p* = 0.019) and had lower EF at discharge (45% vs. 50%; *p* = 0.001) and follow-up (50% vs. 53.5%; *p* = 0.001), respectively. The patients with STEMI were at a lower risk of MACE (0% vs. 7.5%, *p* = 0.030) than those with NSTEMI. Furthermore, the prevalence of comorbidities was significantly different—patients with STEMI had lower rates of DM (19.4% vs. 40%; *p* = 0.006) and hypertension (73% vs. 88.5%; *p* = 0.016), respectively, compared to NSTEMI. All data are presented in Table 7.

### 3.8. Analysis of Haemorrhagic Complications

Haemorrhagic complications were observed in 20 (13%) patients. They were found in 16% of patients initially on Ticagrelor and in 8.3 % of patients initially on Clopidogrel. Further analysis revealed that Clopidogrel, in comparison to Ticagrelor, was significantly more frequently applied in the older population (71.8 vs. 64.2 years; *p* < 0.001) and in the NSTEMI group (52.5% vs. 25 %; *p* < 0.001). On the other hand, Ticagrelor was more frequently applied in the STEMI group (75% vs. 47.5%; *p* < 0.001)

### 3.9. Cardiac Rehabilitation Programme

From the whole group, 48 patients (31.6%) attended a cardiac rehabilitation programme after discharge from the hospital. There were 104 (68.4%) patients who did not take part in this programme. 

Accurate analysis of the group completing the cardiac rehabilitation programme revealed that the duration of index hospitalisation was significantly shorter in comparison to the no rehabilitation group: 5 (IQR 4–5) vs. 7 (IQR 5.75–9) days, *p* < 0.001. Finally, the rehabilitation programme resulted in a reduction in angina pectoris symptom intensification. In the rehabilitation group, 87.5 % was in the CCS class I, while, in the no rehabilitation group, this value totalled 72.1%; *p* = 0.039, respectively. 

## 4. Discussion

The main findings of the current study were: first of all, a significantly higher risk of MACE, especially non-fatal AMI, and a significantly higher risk of hospitalisation regardless of cause among patients with worsening LVEF during follow-up; second of all, significantly more severe symptoms of angina pectoris in the D-LVEF group compared to the P/I-LVEF group; thirdly, we found significant differences among patients with STEMI compared to those with NSTEMI, such as lower LVEF after CR, a lower risk of MACE and less common prevalence of DM. We also noted a correlation between haemorrhagic complications and DAPT with Ticagrelor compared to DAPT with Clopidogrel. There was also an important observation that early rehabilitation allows for a reduction in hospitalisation duration and reduces the severity of angina pectoris. 

Patients with multi-vessel coronary artery disease and AMI represent a therapeutically challenging group of patients. A revascularisation strategy of the culprit artery in STEMI is strictly recommended according to current guidelines. The revascularisation strategy of other critically stenosed arteries or timing of the additional procedure in NSTEMI patients was not unequivocal and has been widely investigated in recent years [6,7]. Finally, it has been documented that complete revascularisation (CR) has beneficial effects in AMI patients. These beneficial effects are generally the result of LVEF value prevention. Low LVEF is a very strong predictor of MACE in AMI patients [8,9]. This is explained by heart failure acceleration, appearance of serious arrhythmias and an increased rate of thromboembolic events. In some studies, it has been shown that the beneficial effect of LVEF improvement was only reached in the group with LVEF < 45% at baseline [10]. Low EF at baseline is more frequently observed in STEMI patients in comparison to the NSTEMI group [11]. This observation was also found in our study. Some investigators have postulated that CR in AMI is sufficient in reducing patient-oriented composite outcome and cardiac death rate in the long-term (three-year) follow-up only among patients with preserved LVEF (>40%) [12]. 

In the present study, we similarly observed that patients with worsening LVEF during follow-up had a significantly higher rate of MACE in comparison to the combined group of patients with LVEF improvement and no-change in LVEF. A low rate of MACE in patients with LVEF improvement can be partially explained by the reduction in thrombosis rate and, in consequence, reduction in death, reMI and rePCI [13]. Oppositely, in patients with worsening LVEF, the similar mechanisms can explain higher rates of re-hospitalisation, which were observed in the present study.

The CR strategy in our study resulted in low MACE rate during follow-up. The median follow-up period was 19 months, which makes this finding reliable because it is postulated that mortality is reduced when CR occurs after 6 months [14]. 

The revascularisation strategy in the present study was based on the CR protocol during index hospitalisation—staged into two PCI procedures separated by a two- to three-day interval. The decision about performing CR was made by the operator according to the angiographic image of the second vessel (i.e., high-grade stenosis, thrombus containing lesion) and clinical characteristics (CAD risk factors). As a result, due to operator’s bias, it can be stated that the final high-risk group of patients was qualified for this study. This explains the higher in-hospital mortality among the CR group (approx. 2–3%), which was documented in other studies [15]. 

What is interesting is that urgent PCI during the follow-up were performed only in men with NSTEMI. In the STEMI group, culprit plaque selection is generally easier than in NSTEMI patients. Moreover, in NSTEMI, the initial angiogram could take more time and more contrast volume to identify culprit lesion [16]. In some ways, it could disturb the proper order of revascularisation during index hospitalisation and could have an impact on the higher rate of urgent PCI in the follow-up. Another confusing factor could be the absence of further assessment regarding myocardium viability before the second stage of the procedure. It could potentially lead to revascularisation with myocardium territory having limited viability.

At the time of discharge, all patients were subjected to dual antiplatelet therapy (DAPT). More haemorrhagic complications were observed in STEMI patients on Ticagrelor in comparison to the NSTEMI and Clopidogrel groups.

The cardiac rehabilitation programme is an integral part of holistic treatment for patients with AMI. In the present study, only ^1^/_3_ of patients had attended the rehabilitation programme. In this subgroup, more patients had minimal angina symptoms in comparison to the patients who did not attend rehabilitation. Moreover, all MACE incidents were observed in the non-rehabilitation group; however, this result was non-significant, *p* = 0.052.

## 5. Limitations

There are several limitations concerning the current study. Usually, we do not perform cardiac echocardiography on patients before admission to STEMI—only during or at the end of hospitalization—and usually, it is one full echo description, admission examination; if it is an indicative examination, often without an entry in the history of the disease, it aims to exclude gross abnormalities such as aortic dissection, assessment of global and regional contractility. This practice also applies to a significant percentage of patients with NSTEMI myocardial infarction, which somewhat limits the interpretation of the data developed and presented in this publication, in particular, regarding the cause-and-effect relationship between late results and left ventricular ejection fraction.

## 6. Conclusions

Complete revascularisation (CR) at baseline (one stage) in patients with AMI and multi-vessel disease is associated with LVEF improvement and MACE rate reduction. Patients with worse LVEF have poor clinical outcome and a higher rate of MACE.

## Figures and Tables

**Table 1:** Baseline characteristics of the study groups.

Characteristic	Study Population (n = 152)	D-LVEF (n = 20)	P/I-LVEF (n = 132)	*p*-Value (D-LVEF vs. P/I-LVEF Group)
Age (years) (mean ± SD)	67.2 ± 10	68.3 ± 9.5	67.0 ± 10.1	0.58 *
Sex (men) (n, (%))	99 (65.1)	10 (50)	89 (67.4)	0.128 **
Hospitalisation (days) (median (IQR))	6 (5–8)	6 (5–11)	6 (5–8)	0.816 ***
Initial diagnosis (n, (%))				0.82 **
STEMI	72 (47.4)	9 (45)	63 (47.7)	
NSTEMI	80 (52.6)	11 (55)	69 (52.3)	
Follow-up (months) (median (IQR))	19 (14–24)	20 (16.5–24)	18 (14–24)	0.32 ***
BMI (kg/m^2^) (median (IQR))	28.5 (25.3–32.1)	32.1 (30.2–34.7)	27.6 (25.1–31.3)	0.032 ***
DM (n, (%))	46 (30.3)	10 (50)	36 (27.3)	0.038 **
Hypertension (n, (%))	120 (81.1)	17 (85)	103 (80.5)	0.63 **
Hypercholesterolemia (n, (%))	103 (68.2)	12 (60)	91 (69.5)	0.397 **
AF (n, (%))	20 (13.2)	0 (0)	20 (15.2)	0.060 **
CKD (n, (%))	14 (9.2)	1 (5)	13 (9.8)	0.485 **
Cigarette smoking (n, (%))	54 (35.5)	3 (15)	51 (38.6)	0.040 **

* Student’s *t*-test, ** Chi square test, *** Mann–Whitney U test. AF; atrial fibrillation, COPD; chronic kidney disease, DM; diabetes mellitus, IQR; interquartile range.

**Table 2:** Comparing LVEF at the time of discharge, LVEF at follow-up and change in LVEF between the D-LVEF group and P/I-LVEF groups.

Characteristic	Study Population (n = 152)	D-LVEF Group (n = 20)	P/I-LVEF Group (n = 132)	*p*-Value (D-LVEF vs. P/I-LVEF Group)
LVEF at discharge (%) (median (IQR))	48.0 (40.0–55.0)	54.5 (49.5–57.0)	46.0 (40.0–54.0)	<0.001 *
LVEF at follow-up (%) (median (IQR))	50.0 (45.0–55.0)	50.0 (41.5–55.0)	50.0 (45.0–55.0)	0.171 *
Change in LVEF (p%) (median (IQR))	+2.0 (0.0–5.0)	−5.0 ((−7.0)–(−2.75))	+4.0 (1.0–5.0)	not applicable

* Mann–Whitney U test. IQR; interquartile range, LVEF; left ventricle ejection fraction.

**Table 3:** Prevalence of MACE and other adverse events among the D-LVEF and P/I-LVEF groups.

Characteristic (n (%))	Study Population (n = 152)	D-LVEF (n = 20)	P/I-LVEF (n = 132)	*p*-Value (D-LVEF vs. P/I-LVEF Group)
MACE	6 (4)	3 (15)	3 (2.3)	0.031 *
Death	2 (1.3)	1 (5)	1 (0.8)	0.247 *
Non-fatal AMI	2 (1.3)	2 (10)	0 (0)	0.017 *
Non-fatal stroke	2 (1.3)	0 (0)	2 (1.5)	1.000 *
Urgent PCI	4 (2.6)	2 (10)	2 (1.5)	0.084 *
Planned PCI	7 (4.6)	1 (5)	6 (4.5)	1.000 *
Hospitalisation	38 (25)	9 (45)	29 (22)	0.027 **
Haemorrhagic complications	20 (13.2)	3 (15)	17 (12.9)	0.729 *
Local complications	9 (5.9)	1 (5)	8 (6.1)	1.000 *

* Fisher’s exact test, ** Chi square test. AMI; acute myocardial infarction, MACE; major adverse cardiac events, PCI; percutaneous coronary intervention.

**Table 4:** Univariate analysis of MACE predictors.

Variable	OR	95% CI	*p*-Value
Urgent PCI, yes vs. no	35.75	3.60–373.07	0.004
Hospitalisation need regardless of cause, yes vs. no	6.53	1.22–48.58	0.028
EF worsening, yes vs. no	7.53	1.31–43.64	0.025
Glucose, mmol/L	1.04	1.01–1.07	0.024
TGL, mmol/L	1.37	1.04–1.86	0.027
HDL-cholesterol, mmol/L	0.037	0.0009–0.77	0.031

CI; confidence interval, HDL-cholesterol; high-density lipoprotein cholesterol; MACE; major adverse cardiac events, PCI; percutaneous coronary intervention, OR; odds ratio.

**Table 5:** Diabetes mellitus, risk of MACE and other adverse events.

Characteristic (n (%))	Diabetic (n = 46)	Non-Diabetic (n = 105)	*p*-Value
MACE	3 (6.5)	3 (2.9)	0.369 *
Death	1 (2.2)	1 (0.9)	0.515 *
Non-fatal AMI	1 (2.2)	1 (0.9)	0.515 *
Non-fatal stroke	1 (2.2)	1 (0.9)	0.515 *
Urgent PCI	2 (4.3)	2 (1.9)	0.585 *
Planned PCI	1 (2.2)	6 (5.7)	0.676 *
Hospitalisation	20 (43.5)	18 (17)	<0.001 **
Haemorrhagic complications	3 (6.5)	17 (16)	0.126 *
Local complications	3 (6.5)	6 (5.7)	1.000 *

* Fisher’s exact test, ** Chi square test. AMI; acute myocardial infarction, MACE; major adverse cardiac events, PCI; percutaneous coronary intervention.

**Table 6:** Relationship between LVEF change and CCS class grade in the follow-up.

CCS (n (%))	Study Population (n = 152)	D-LVEF (n = 20)	P/I-LVEF (n = 132)	*p*-Value (D-LVEF vs. P/I-LVEF Group)
I	117 (77)	11 (55)	106 (80.3)	0.006 *
II	28 (18.4)	5 (25)	23 (17.4)	0.208 *
III	6 (3.9)	4 (20)	2 (1.5)	<0.001 *
IV	1 (0.7)	0 (0)	1 (0.8)	0.348 *

* Chi square post-hoc test, *p* < 0.001.

**Table 7:** Differences between STEMI and NSTEMI patients.

Characteristics	STEMI (n = 72)	NSTEMI (n = 80)	*p*-Value
Age (years) (mean ± SD)	65.5 ± 9.8	69 ± 9.9	0.019 *
EF at discharge (%) (median, (IQR))	45 (38–50)	50 (45–55)	<0.001 **
EF follow-up (%) (median, (IQR))	50 (44–54)	53.5 (48–57)	<0.001 **
MACE (n, (%))	0 (0)	6 (7.5)	0.03 ***
Death	0 (0)	2 (2.5)	0.498 ***
Non-fatal AMI	0 (0)	2 (2.5)	0.498 ***
Non-fatal stroke	0 (0)	2 (2.5)	0.498 ***
Urgent PCI	0 (0)	4 (0)	0.122 ***
Planned PCI	4 (5.6)	3 (3.8)	0.708 ***
Hospitalisation	18 (25)	20 (25)	1.000 ****

* Student’s *t*-test, ** Mann–Whitney U test, *** Fisher’s exact test, **** Chi square test. IQR; interquartile range, SD; standard deviation.

## Data Availability

Further information can be obtained from the corresponding author.
